# Mediastinal syndrome from plasmablastic lymphoma in human immunodeficiency virus and human herpes virus 8 negative patient with polycythemia vera: a case report

**DOI:** 10.1186/s13256-016-1183-1

**Published:** 2017-03-21

**Authors:** Massimo Cajozzo, Vincenzo Davide Palumbo, Salvatore Buscemi, Giuseppe Damiano, Ada Maria Florena, Daniela Cabibi, Francesco Raffaele, Antonino Alessio Anzalone, Federica Fatica, Gerlando Cocchiara, Salvatore Dioguardi, Antonio Bruno, Francesco Paolo Caronia, Attilio Ignazio Lo Monte

**Affiliations:** 1 Department of Surgical, Oncological and Stomatological Disciplines, University of Palermo, Via Del Vespro 129, 90127 Palermo, Italy; 2 Euro-Mediterranean Institute of Science and Technology (IEMEST), Palermo, Italy; 3 Department of Science for Health Promotion and for Mother and Child “G. D’Alessandro”, University of Palermo, Palermo, Italy; 4 Department of Diagnostic Medicine and Prevention, S. Orsola-Malpighi Hospital, University of Bologna, Bologna, Italy; 5 Mediterranean Oncological Institute (IOM), Catania, Italy

**Keywords:** Case report, Fine-needle aspiration biopsy, Hematology, Rare clinical case, Thoracic surgery

## Abstract

**Background:**

Plasmoblastic lymphoma is a rare and aggressive subtype of diffuse large B cell lymphoma, which occurs usually in the jaw of immunocompromised subjects.

**Case presentation:**

We describe the occurrence of plasmoblastic lymphoma in the mediastinum and chest wall skin of an human immunodeficiency virus-negative 63-year-old Caucasian man who had had polycytemia vera 7 years before. At admission, the patient showed a superior vena cava syndrome, with persistent dyspnoea, cough, and distension of the jugular veins. Imaging findings showed a 9.7 × 8 × 5.7 cm mediastinal mass. A chest wall neoformation biopsy and ultrasound-guided fine-needle aspiration biopsy of the mediastinal mass allowed diagnosis of plasmoblastic lymphoma and establishment of an immediate chemotherapeutic regimen, with rapid remission of compression symptoms.

**Conclusions:**

Plasmoblastic lymphoma is a very uncommon, difficult to diagnose, and aggressive disease. The presented case represents the first rare mediastinal plasmoblastic lymphoma in a human immunodeficiency virus-/human herpesvirus-8-negative patient. Pathologists should be aware that this tumor does appear in sites other than the oral cavity. Fine-needle aspiration biopsy is a low-cost, repeatable, easy-to-perform technique, with a high diagnostic accuracy and with very low complication and mortality rates. Fine-needle aspiration biopsy could represent the right alternative to surgery in those patients affected by plasmoblastic lymphoma, being rapid and minimally invasive. It allowed establishment of prompt medical treatment with subsequent considerable reduction of the neoplastic tissue and resolution of the mediastinal syndrome.

## Background

By World Health Organization (WHO) classification, plasmoblastic lymphoma (PBL) is considered to be a new subtype of diffuse large B cell lymphoma (DLBCL) with distinct blastic morphology, antigenic phenotyping data indicating plasmacytic differentiation (CD20^−^, CD45^−^, CD79a^+^, and VS38c^+^), and clinical presentation favoring extramedullary sites, particularly the oral cavity and the mucosa of the jaw [1]. PBL is associated with human immunodeficiency virus (HIV) and Epstein–Barr virus (EBV) co-infection; its incidence has increased since the introduction of the highly active anti-retroviral therapy (HAART) [2]. Based on morphology alone, the differential diagnosis would include lymphoblastic lymphoma, anaplastic plasmacytoma, plasmablastic variant of BCL and human herpesvirus-8 (HHV8)-associated PBL. Although most of the tumors show plasmablastic morphology, express plasma cell antigens, are EBV-positive and have a high proliferative index, they still remain a heterogeneous group with varying clinical presentation and hence differing treatment approaches. The clinical course of PBL is characteristically aggressive. It is generally associated with early dissemination and poor response to therapy and has a reported median overall survival time of 15 months [3]. Currently, treatment responses are usually partial and temporary; however, prolonged and durable responses to chemotherapy have been reported [4–6]. In one small series, early recognition of this pathological entity, more aggressive management and better HIV disease control led to improved outcomes with a median follow-up on 22 months and the median survival not yet reached at the time of reporting [7]. This is the first reported case of mediastinal PBL. Interestingly, the patient affected was HIV-/HHV8-negative. In this particular case, PBL had a metachronous lesion of the chest wall. A prompt detection and mini-invasive approach allowed our patient to recover within a few cycles of chemotherapy.

## Case presentation

A 63-year-old Caucasian man was referred to our hospital with a 3-month history of dyspnoea, hacking cough, and distended jugular veins.

Seven years before, a complete blood cell count had revealed polyglobulia. The subsequent bone marrow biopsy showed a cellularity of 70%, with trilinear expansion; the myelo-erythroid ratio was 5:1, with preserved maturation. The proliferative megakaryocytic series were slightly polymorphic, with little tendency to cluster aggregation. The histology was highly suspicious for an initial phase of polycythemia vera (Vasquez disease). After diagnosis, our patient was placed on oral administration of 1,4-bis (3-bromopropionyl) piperazine 25 mg and hydroxyurea 500 mg. Three years later, JAK2 V617F mutation was found. Our patient did not refer for any further relevant medical issue.

**Fig. 1:**
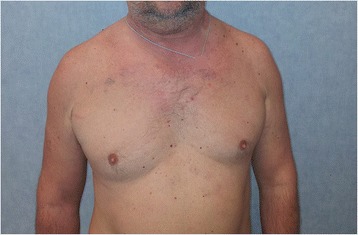
Facial rash, periorbital and facial edema, bilateral disappearance of the supraclavicular fossae

On physical examination, our patient showed facial rash, periorbital and facial edema, distension of the jugular veins, bilateral disappearance of his supraclavicular fossae, mild ptosis especially on the right side, and a chest wall formation, just upon the projection of the sixth right rib, of hard consistency (Fig. 1). A neurological examination was negative. Our patient showed signs neither of laryngeal and phrenic nerve involvement (dysphonia, hiccough) nor of abnormal sympathetic response (Bernard-Horner syndrome).

Blood test results showed relative (17.5%) and absolute (1.57*10^3^/μL) lymphocytosis; his lactate dehydrogenase (LDH) was 423 U/L; C-reactive protein (CRP) 1.8 mg/dL; fibrinogen 414 mg/dl; immunoglobulin A (IgA) 549 mg/dL; erythrocyte sedimentation rate (ESR) was 27 mm; β_2_-microglobulin was 2.6 mg/dL. Our patient was negative for hepatitis C (HCV), HHV8 and HIV, but positive for hepatitis B (HBV) (HBc IgG: 627 mIU/mL) and EBV.

**Fig. 2:**
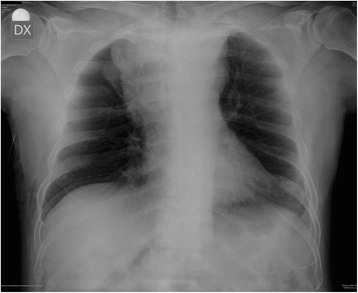
Chest X-ray; antero-posterior view. Upper mediastinal widening due to a mediastinal mass (10 cm × 6 cm), more evident on the right side, with polycyclic margins; the angles with the mediastinal contour are obtuse. The hilar vessels cannot be seen through the mass (“hilum overlay sign” absent). The paravertebral line can be recognized. These two findings confirm that this mass is located in the superior-anterior-middle mediastinum. Trachea is deviated laterally to the right side by the mediastinal mass. At the right inferior chest wall subcutaneous tissue mass can be detected

**Fig. 3:**
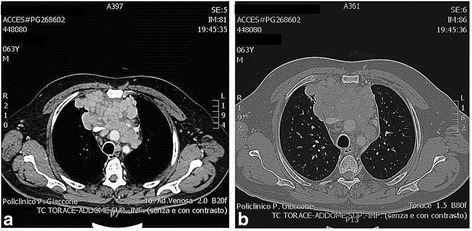
Chest contrast-enhanced computed tomography scan. **a** Mediastinal window and **b** lung window: large mediastinal mass (axial: 9.7 × 5.7 cm; longitudinal: 8 cm) formed by multiple confluent lymph nodes with a heterogeneous contrast enhancement that involves the anterior-middle mediastinum. The mass is compressing the jugular vein and the superior cava vein

A chest X-ray detected a bilateral flaring, especially in the upper right side; the trachea was deviated on the right (Fig. 2). A total body computed tomography (CT) scan, performed a few days after admission, showed a polylobed solid heteroplasia to the anterior-superior mediastinum, iso-hypodense after contrast, of 9.7 cm in its transverse diameter, 8 cm in its cranio-caudal diameter and 5.7 cm in its antero-posterior diameter. The heteroplasia compressed the right jugular vein and the superior vena cava. The visible chest wall formation showed the same radiological characteristics as the mediastinal one (Fig. 3). No lesion was found in our patient’s abdomen or skull.

**Fig. 4:**
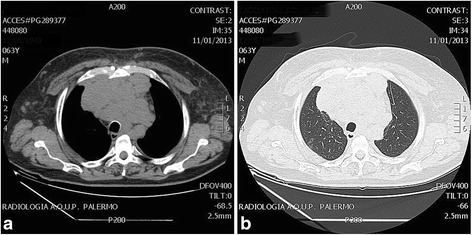
Chest computed tomography scan. **a** Mediastinal window and **b** lung window: mediastinal mass 1 month later (axial 12 × 7.5 cm; longitudinal 14 cm). The size of the heteroplasia increased about 10%

One month after a total body CT scan, another chest CT scan was performed: in this case, the mass showed increased dimensions, being 12 cm in transverse diameter, 14 cm in cranio-caudal diameter and 7.5 cm in antero-posterior diameter (Fig. 4).

**Fig. 5:**
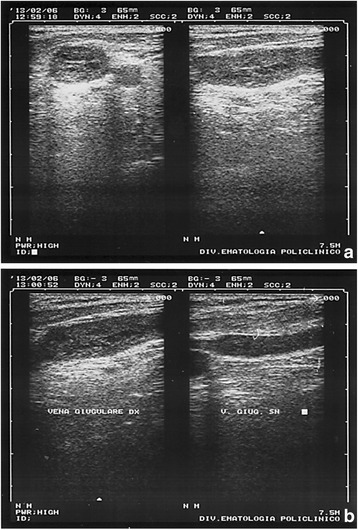
Jugular vein echography. **a** Right internal jugular vein thrombosis, in transverse and longitudinal sections. A heterogeneous echogenic material fills and distends the right internal jugular vein; **b** comparison of the right and left internal jugular veins. Ultrasonography shows different diameter between the two vessels

One month later, a neck ultrasound (US) scan showed a thrombosis of the right internal jugular vein with signs of recanalization, despite previous anticoagulation therapy. The left internal jugular vein was patent (Fig. 5).

**Fig. 6:**
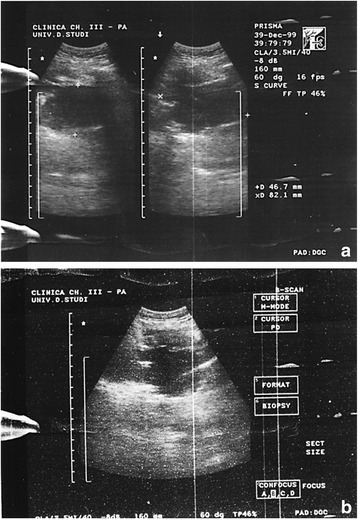
Intercostal chest echography (second right intercostal space). **a** Ultasound image shows a thin-walled hypo-anechoic formation in the anterior mediastinum (46.7 mm in anteroposterior and 82.1 mm in laterolateral diameter); **b** fine-needle aspiration biopsy

A diagnostic mediastinal ultrasound (US)-guided fine-needle aspiration biopsy (FNAB) was performed with local anesthesia, using a low-frequency probe (3.5 MHz), (Fig. 6a). The mediastinal mass was showed as a hypo-/anechoic formation at the depth of 35 mm, with a 46.7 mm antero-posterior diameter and 82.1 mm transverse diameter (Fig. 6b). Contemporaneously, the chest wall neoformation was resected, always under local anesthesia with lidocaine 20% and naropine 1%. No complication occurred during and after surgery. Both samples were sent to pathologists.

**Fig. 7:**
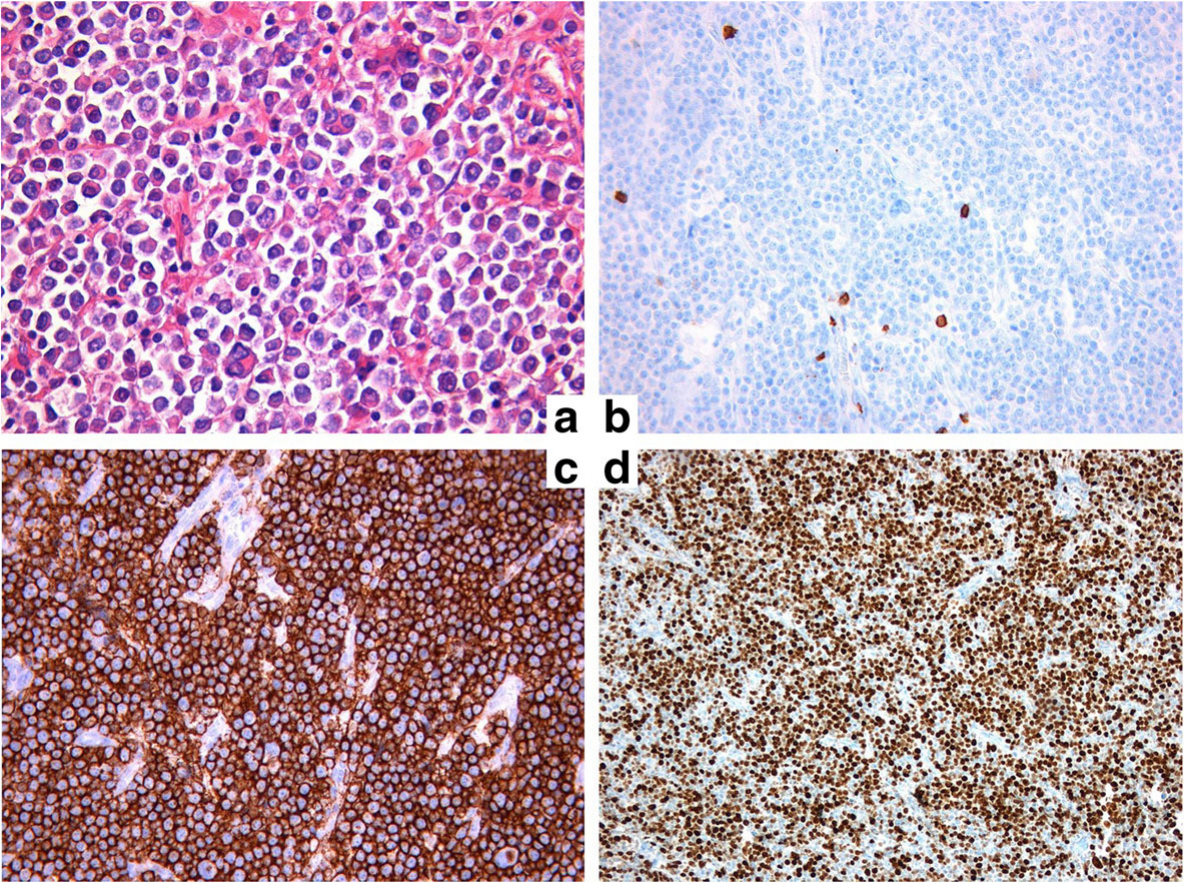
Fine-needle aspiration biopsy histology. **a** Monomorphic lymphoid proliferation composed of large-size cells with evident nucleulus, typical of a high-grade malignant non-Hodgkin’s B cell lymphoma; **b** immunophenotypic LCA^+^, CD20‾; **c** immunophenotypic CD79a^+^, MUM1^+^, CD38^+^, CD138^+^; **d** immunophenotypic κ^+^, λ‾, CD56‾, CD30‾, ALK1‾, CD3‾, CD5‾, CD10‾, bcl-2‾, bcl-6‾, TdT‾, LMP1‾, Ki-67: 85%

Histological examination of the mediastinal FNAB and the chest wall biopsy gave the same results: resected tissue widely occupied by a monomorphic lymphoid proliferation (Fig. 7a) composed of large-size cells with evident nucleoli, typical of a high-grade non-Hodgkin’s B cell lymphoma [8, 9]. Morphological and immunophenotypic findings (Fig. 7b, c, d) (PanCK, CK20, chromogranin, NSE were also negative) were compatible with the diagnosis of PBL.

Our patient was pharmacologically treated with EPOCH, a combined therapy of intravenous etoposide (50 mg/m^2^/day), vincristine (0.4 mg/m^2^/day), and doxorubicin (10 mg/m^2^/day) for 96 hours with bolus doses of cyclophosphamide (750 mg/m^2^/day) and oral prednisone (60 mg/m^2^/bid) [10]. Treatment response was good, with a prompt regression of symptoms after the first cycle. Our patient received five cycles of combination chemotherapy with good response. After 10 years, our patient is still alive and healthy with no clinical or radiological evidence of recurrent or residual disease.

## Discussion

PBL is characterized by its predilection of involving the oral cavity of HIV-positive individuals as originally described [1]. Following the first report, a number of cases have been reported in extraoral sites, in HIV-positive cases. The most commonly affected sites are the gastrointestinal tract, lymph nodes, and skin [3, 11–14]. A similar pattern is seen in patients with HIV-negative PBL, with the oral cavity and gastrointestinal tract being the most commonly involved sites [15]. The frequency of oral involvement is higher in HIV-positive (58%) than in HIV-negative patients (16%) [16]. Other less common extraoral sites include the central nervous system [17, 18], paranasal sinuses [18, 19], lungs [19–21], liver [21], and testes [12, 22]. Bone marrow involvement has been reported at 30% in both HIV-positive and HIV-negative patients [16]. PBL has also been documented to arise from longstanding sacrococcygeal cysts in HIV-positive persons [4]. In a literature review of 228 patients with PBL, 157 patients (69%) were HIV-positive and 71 (31%) were HIV-negative [16]; among HIV-negative patients, 33% of the patients had some form of immunosuppression, most often solid organ transplantation or steroid therapy [23]. The remainder of the HIV-negative patients were apparently immunocompetent. In a recent case series from Korea, none of the patients reported showed evidence of immunosuppression [24]. In this rare case, our patient had no evidence of HIV or HHV-8 infections, and he was apparently healthy. We can postulate that PBL developed years before, probably when he had his cycles of chemotherapy for his polycythemia. The size of the mediastinal mass (9.7 × 8 × 5.7 cm) could confirm this hypothesis. In such a case, an accurate histological evaluation of the endothoracic mass is crucial to establish the optimal medical treatment, in particular when a morbid surgery is the alternative [20]. Excisional biopsy should be the gold standard; however, when the site of the disease is difficult to access, as in this case, core needle biopsy and FNAB may be performed in conjunction with appropriate ancillary techniques for the diagnosis and differential diagnosis. In fact, the histopathological features are frequently ambiguous, thus rendering the correct diagnosis quite difficult. This neoplasm may be confused with the plasmablastic type of plasma cell myeloma; however, the absence of serum monoclonal protein and lack of significant bone marrow involvement may argue against this diagnosis [1]. In addition, PBL is almost entirely composed of blasts with numerous mitotic figures. These features are not typical for plasma cell myeloma. The plasma cell markers VS38c, CD38, multiple myeloma oncogene-1 (MUM1), and CD138 (syndecan-1) seem to be almost universally expressed [3, 9, 25]. PBL is characterized by a high proliferation index reflected by Ki67 expression, usually > 80%. Immunophenotypically, the neoplastic cells lacked B cell-associated antigens (CD20, CD45RA, and CD79a). T cell-associated antigens (CD3 and CD45RO) as well as leukocyte common antigen (CD45) were also absent. The neoplastic cells expressed plasma cell-associated antigens CD138 and VS38c. Transthoracic FNAB allowed making the right diagnosis thus saving the patient uncomfortable, more invasive, procedures, like in other similar cases [20]. Both biopsies, mediastinal and cutaneous, were performed under local anesthesia, and our patient was allowed to start his chemotherapy promptly. An aggressive medical therapy resolved, just after the first cycle and without invasive surgery, our patient’s superior vena cava syndrome (cutaneous rash, right palpebral ptosis, dyspnoea, cough) [26].

PBL is a therapeutic challenge with a clinical course characterized by a high rate of relapse and death. The prognosis is generally poor, with most patients dying within 2 years from initial presentation, and long-term survivors are very few. Patients carrying the *MYC/IgH* gene rearrangement have been shown to have a very poor median overall survivor of only 3 months.

A standard therapy has not yet been established. Treatment usually consists of chemotherapy with or without consolidation radiation and hematopoietic stem cell transplantation [27]. Various chemotherapy regimens including cyclophosphamide, doxorubicin, vincristine, and prednisone (CHOP), R-CHOP, and cyclophosphamide, vincristine, doxorubicin, high-dose methotrexate/ifosfamide, etoposide, and high-dose cytarabine (CODOX-M/IVAC) are also possible options [10, 28]. Patients with PBL who were not treated with chemotherapy invariably died with a median survival of 3 months [16]. Due to disappointing response and survival rates, the National Comprehensive Cancer Network (NCCN) guidelines recommend against CHOP in favor of more intensive regimens, such as intravenous EPOCH, cyclophosphamide, vincristine, doxorubicin, and dexamethasone (hyper-CVAD), or CODOX-M/IVAC [10]. One of the newest therapeutic options for PBL is bortezomib, which is a proteasome inhibitor and a cornerstone in myeloma and relapsed or refractory mantle cell lymphoma therapy [29]. Some studies have reported that the proteasome inhibitor bortezomib alone or in combination with chemotherapy may have an antitumor effect in PBL or overcoming the typical chemoresistance of this disease. For the same reason, the use of lenalidomide has been reported in PBL [30]. In the presented case, the EPOCH scheme brought the best outcome, with a rapid response, a prompt resolution of compression symptoms and a final complete recovery.

## Conclusions

PBL is a very uncommon, difficult to diagnose, and aggressive disease. The presented case represents the first rare mediastinal PBL in a HIV-/HHV8-negative patient. Pathologists should be aware that this tumor does appear in sites other than the oral cavity. Because of its cohesive histologic appearance, this tumor can be misinterpreted as being a nonlymphoid tumor, particularly with the leukocyte common antigen negativity that is typical of this neoplasm. In a small biopsy specimen, the diagnosis can be even more problematic and challenging for the pathologist.

A timely detection and a prompt treatment is mandatory to avoid life-threatening consequences. The FNAB could be a low-cost, repeatable, easy-to-perform technique, with a high diagnostic accuracy and with very low complication and mortality rates. FNAB could represent the right alternative to surgery in those patient affected from PBL, being rapid and mininvasive. It allowed establishment of a prompt medical treatment with a subsequent considerable reduction of the neoplastic tissue and the resolution of the mediastinal syndrome.

## Abbreviations

CHOP: cyclophosphamide, doxorubicin, vincristine, prednisone
CODOX-M/IVAC: cyclophosphamide, vincristine, doxorubicin, high-dose methotrexate/ifosfamide, etoposide, high-dose cytarabine
CT: computed tomography
DLBCL: diffuse large B cell lymphoma
EBV: Epstein–Barr virus
FNAB: fine-needle aspiration biopsy
HAART: highly active anti-retroviral therapy
HHV8: human herpesvirus-8
HIV: human immunodeficiency virus
NCCN: National Comprehensive Cancer Network
PBL: plasmoblastic lymphoma
US: ultrasonography
WHO: World Health Organization
